# Joint modeling of longitudinal changes of blood pressure and time to remission of hypertensive patients receiving treatment: Bayesian approach

**DOI:** 10.1371/journal.pone.0281782

**Published:** 2023-02-16

**Authors:** Frezer Tilahun Getaneh, Lijalem Melie Tesfaw, Zelalem G. Dessie, Muluwerk Ayele Derebe

**Affiliations:** 1 Department of Statistics, Mekdela Amba University, Mekdela, Ethiopia; 2 Department of Statistics, Bahir Dar University, Bahir Dar, Ethiopia; 3 Epidemiology and Biostatistics, School of Public Health, Faculty of Medicine, University of Queensland, Queensland, Australia; 4 School of Mathematics, Statistics and Computer Science, University of KwaZulu-Natal, Durban, South Africa; Osaka University of Pharmaceutical Sciences, JAPAN

## Abstract

**Introduction:**

Hypertension is a widespread condition when the blood’s force on the artery walls is extremely high to develop adverse health effects. This paper aimed to jointly model the longitudinal change of blood pressures (systolic and diastolic) and time to the first remission of hypertensive outpatients receiving treatment.

**Methods:**

A retrospective study design was used to collect appropriate data on longitudinal changes in blood pressure and time-to-event from the medical charts of 301 hypertensive outpatients under follow-up at Felege Hiwot referral hospital, Ethiopia. The data exploration was done using summary statistics measures, individual profile plots, Kaplan-Meier plots, and log-rank tests. To get wide-ranging information about the progression, joint multivariate models were employed.

**Results:**

A total of 301 hypertensive patients who take treatment was taken from Felege Hiwot referral hospital recorded between Sep. 2018 to Feb. 2021. Of this 153 (50.8%) were male, and 124 (49.2%) were residents from rural areas. About 83(27.6%), 58 (19.3%), 82 (27.2%), and 25 (8.3%) have a history of diabetes mellitus, cardiovascular disease, stroke, and HIV respectively. The median time of hypertensive patients to have first remission time was 11 months. The hazard of the patient’s first remission time for males was 0.63 times less likely than the hazard for females. The time to attain the first remission for patients who had a history of diabetes mellitus was 46% lower than for those who had no history of diabetes mellitus.

**Conclusion:**

Blood pressure dynamics significantly affect the time to the first remission of hypertensive outpatients receiving treatment. The patients who had a good follow-up, lower BUN, lower serum calcium, lower serum sodium, lower hemoglobin, and take the treatment enalapril showed an opportunity in decreasing their blood pressure. This compels patients to experience the first remission early. Besides, age, patient’s history of diabetes, patient’s history of cardiovascular disease, and treatment type were the joint determinant factors for the longitudinal change of BP and the first remission time. The Bayesian joint model approach provides specific dynamic predictions, wide-ranging information about the disease transitions, and better knowledge of disease etiology.

## Background

When the blood’s strain on the arterial walls is excessively high, a common ailment known as hypertension results [1, 2]. According to a World Health Organization report [3], most hypertensive individuals reside in impoverished nations with underdeveloped healthcare infrastructure and low levels of public awareness. Around the world, hypertension is more or less common [2, 4]. Worldwide, the prevalence of hypertension has been increasing at an increasing rate from 2000 to date. About 972 million (26.4%) people were exposed to hypertension in 2000. These are anticipated to increase to 1.54 billion (29.2%) people who will have hypertension in 2025 [5].

Poor hypertension management and a high incidence of hypertension are major risk factors for heart disease in underdeveloped nations like Ethiopia. Studies in Africa [4–6] such as Nigeria, Tanzania, Kenya, and Ethiopia reported the prevalence of hypertension ranged from 10.1% in Southern Ethiopia to 23.7% in Tanzania. Study findings in Ethiopia reported that the prevalence of hypertension was as high as 28.3 to 30.0% in Gondar [6] and Addis Ababa [7] respectively.

The high prevalence rates of hypertension are influenced by a variety of socioeconomic, demographic, biological, and behavioral factors. The prevalent significant predictors are drinking alcohol, genetics, a lack of access to health care, an unbalanced diet, and inactivity [5, 8]. In low and middle-income countries there is a high incidence of hypertension in comparison with developed countries. Although there have been numerous publications published on hypertension globally, there are still few in underdeveloped nations like Ethiopia.

For studying the development or evolution of patients over time, or to gauge the impact of treatment on patients, many clinical studies use repeated measurements data or longitudinal data. Following therapy modifications over time, the average systolic and diastolic blood pressure biomarkers of hypertensive patients were primarily reported [9]. A separate longitudinal study conducted by [10] aims to assess the trends of systolic and diastolic BP over time using a bivariate linear mixed model.

At the follow-up appointment, conversion to normotensive status (SBP<140 mmHg and DBP<90 mmHg with no usage of antihypertensive medication) was considered remission of hypertension [11, 12].

Numerous research [2, 4, 8] employed the linear mixed-effects model (LMM) for longitudinal data and parametric or semi-parametric models for survival data to analyze longitudinal and survival data separately. The joint model, however, has received little focus and is quite constrained in how it simultaneously predicts the longitudinal and time-to-event components. The joint model of the time-to-event and longitudinal outcome is better than the separate one [13, 14]. Joint modeling is suitable for extensive improvements in estimations compared to longitudinal and survival models separately [15]. Joint models have been employed in studies [16, 17] utilizing the frequentist approach, where the parameters are obtained by maximizing the likelihood. However, it is more beneficial to implement a Bayesian approach as it is more upfront, and historical data can be incorporated easily into the inference procedure [13]. Therefore, this study aimed to jointly model the longitudinal change of blood pressures (SBP and DBP) and time to the first remission of hypertensive outpatients receiving treatment via the Bayesian approach. The results will be helpful for the patients to have awareness of ways to control blood pressure changes, for health professionals and policymakers, and it will help as a reference for other researchers.

## Methods

### Study area

The study was conducted at Felege Hiwot Referral Hospital (FHRH), Bahir Dar, Ethiopia. The Hospital gives service to people who are living in Northwest Ethiopia. It is away 564 KM from Addis Ababa, the capital city of Ethiopia [16].

### Study design and sample size determination

A retrospective study design was implemented to extract all the necessary information from hypertensive patients receiving treatment in the hospital. The data was collected from all hypertensive patients who received treatment from their medical charts under the follow-up from September 2018 to February 2021. All the socio-demographic and medical data of the hypertensive patients under the follow-up was obtained from their respective registry cards.

The sample size determination used in this study was based on [17] which is given by:

$$n=\frac{4{\left(Z_{\frac{\alpha }{2}}-Z_{1-\beta }\right)}^{2}}{p*\theta_{R}^{2}}=\frac{4{\left(Z_{\frac{\alpha }{2}}+Z_{\beta }\right)}^{2}}{p*\theta_{R}^{2}}$$

Where n is the required number of patients to be included in the study, *α* is the level of significance and we take the value of 5%, 1−*β* is the power of the test which equals 80%, p is the probability of patients expected to have good control of blood pressure (first remission) with the value of 0.504, and *θ*_*R*_ = ln(*ψ*) is the log of the hazard ratio of patients to remission who had no DM with a value of 0.4121 based on [18]. The total sample size considered in this study was 301.

Inclusion and Exclusion Criteria: All hypertensive outpatients whose age is above 15 years old and received treatment and had more than one visit from their medical charts under the follow-up from September 2018 to February 2021, were included in the study.

#### Ethics approval and consent to participant

Ethical clearance was obtained from the ethical review of Felege Hiwot Referral Hospital with the Science College Research and Community Service Committee (SCRCSC) of Bahir Dar University, with reference number SCRCSC/102/02/13. As the data was obtained from a secondary source informed consent was not required by the ethical review board.

### Variables in the study

The variables involved under this study were determined based on the patient registry card. Variables which are not fully accessible in the patient registry card were excluded.

### Dependent variables

The dependent variables; bivariate longitudinal measure SBP and DBP in mmHg, and time to the first remission of hypertension outpatients. Remission of hypertension was defined as conversion to normotensive status (SBP < 140 mmHg and DBP < 90 mmHg and no use of antihypertensive medication) at follow-up visit, and reduction of ≥30% of antihypertensive medications while maintaining an office SBP <120 mm Hg at 12 months [19].

### Independent variables

The list of independent variables and their description, categories, and coding were presented in Table 1.

**Table 1:** Independent variables and coding.

S/No.	Variables	Description	Categories and code
1.	Visit time	Observation times (in month)	Continuous
2.	Age	Baseline age of patients (in years)	Continuous
3.	Sex	Sex of patients	Female = 0, Male = 1
4.	Residence	Residence of patients	Urban = 0, Rural = 1
5.	Cholesterol	Cholesterol level	Continuous
6.	BCL	Blood cholesterol level (in mg/dl)	Continuous
7.	GBL	Blood glucose level (in mg/dl)	Continuous
8.	BUN	Blood urea nitrogen (in mg/dl)	Continuous
9.	Creatinine	A creatinine level of patients	Continuous
10.	Serum	Serum electrolytes levels (in mmol/l) of calcium, sodium, potassium, and chlorine	Continuous
11.	Regimen	Drug therapy regimen of patients	Monotherapy = 0, two drug therapy = 1, three or more drug therapy = 2
12.	Treatment	Patients took drug type	Enalapril = 0, Nifedipine = 1, Enalapril + Nifedipine, 3 = Others
13.	Comorbidities	Presence of history of diabetes mellitus (DM), cardiovascular disease (CVD), chronic kidney disease (CKD), and stroke.	Yes = 0, No = 1
14.	HIV	HIV status of patients	Positive = 0, Negative = 1

### Statistical methods

The data were explored using individual profile plots, mean profile plots, and the Kaplan-Meier curve. Then, the longitudinal measures from SBP and DBP were taken within follow-up, and the time to the first remission of patients was analyzed separately to identify the determinant factors for both models, and jointly to assess the influence of the longitudinal change of SBP and DBP on survival time till to the first remission among hypertension outpatients via Bayesian approach.

### Bayesian joint longitudinal-survival models

Modeling the joint longitudinal-survival data using the Bayesian approach is more effective as the parameter estimation computation is easier and flexible as compared to frequents approach [13]. Besides, in Bayesian analysis, the prior information of the data can be easily incorporated. For the vector of fixed effects of the longitudinal submodel we assume multivariate normal priors with mean zero and variance 1000. For the regression coefficients *γ* of the survival model, we assume independent normal priors with mean zero and variance 1000. The association parameters *α* have a global-local ridge-type shrinkage prior. More specifically, for the sth element *α*, we assume:

$$\rho_{s}∼N(0,\tau \psi_{s}),$$

$$\tau^{-1}∼Gamma(0.1,0.1)$$

$${\psi_{s}}^{-1}∼Gamma(1,0.01)$$

The global smoothing parameter *τ* has sufficient mass near zero to ensure shrinkage, while the local smoothing parameter *ψ*_*s*_ allows individual coefficients to attain large values. The motivation for using this type of prior distribution, in this case, is that we expect the different terms behind the specification of *f*(.) (postulate that the hazard of an event at time *t* may be associated with the underlying level of the biomarker at the same time point, the slope of the longitudinal profile at *t* or the accumulated longitudinal process up to *t*.) to be correlated and many of the corresponding coefficients to be non-zero [20].

The goodness of fit of the joint longitudinal survival model was checked using the novel decomposition of AIC and BIC [21].

In the Bayesian approach, the posterior distribution of the data is obtained from the conjugate of the likelihood of the current data and some prior distribution. The two components sub-models of the joint model are the longitudinal measurement model and the time to remission model which share one or more common parameters.

The joint posterior distribution for parameters *θ* is then given by [22]:

$$f(\theta ,b_{i}|Y,T)=\frac{f(y,T|\theta ,b)\pi (\theta )\pi (b)}{\int f(y,T|\theta ,b)\pi (\theta )\pi (b)d\theta \;db}$$

where *f*(*θ*,*b*_*i*_|*Y*,*T*) is the posterior probability distribution, *f*(*y*,*T*|*θ*,*b*) is the likelihood function and *π*(*θ*), *and π*(*b*) is the prior probability distribution. In this framework, all the inference is carried out using the posterior distribution. In this study MCMC algorithm was used to draw samples from the posterior distribution [23, 24].

#### Longitudinal sub-model

The longitudinal responses ***Y***_***ij***_, are inherently measured intermittently with an error. To incorporate the subject-specific trajectory and estimate the parameters linear mixed model framework was utilized [25]. Therefore it can be defined as:

$$Y_{ij}(t_{ij})=M_{i}(t_{ij})+\epsilon_{ij}(t_{ij}),\epsilon_{ij}(t_{ij})∼N(0,\sigma_{\epsilon }^{2})$$

$$M_{i}(t_{ij})={X_{i}(t_{ij})}^{\prime }\beta +{{Z_{i}(t_{ij})}^{\prime }b}_{i}+u_{i}^{\prime }\delta ,b_{i}∼N(0,\sum )$$

Where ***β*** is a vector of fixed effects of ***X***_***i***_(***t***_***ij***_) time-varying covariate matrix**, b*i*** is a vector of random slope effects of ***Z***_***i***_(***t***_***ij***_) time-varying covariate matrix. ***u***_***i***_ is a vector of time-invariant covariates contained in some larger set ***U***_***i***_(***u***_***i***_∈***U***_***i***_), and corresponding regression coefficients, ***δ***. The model assumed the measurement error, *ϵ*_*ij*_(*t*_*ij*_) is normally distributed with variance $\sigma_{\epsilon }^{2}$, and is independent of the random effects, and that *cov*(*ϵ*_*ij*_(*t*_*ij*_), *ϵ*_*ik*_(*t*_*ik*_)) = 0 (*where j*≠*k*).

#### Survival sub-model

*PHs survival sub-model*. The joint model aims to link the component processes together using shared parameters. Let *m*_*i*_(*t*) indicate the true unknown patient-specific longitudinal trajectory, and let *M*_*i*_(*t*) = {*m*_*i*_(*s*), 0≤*s*≤*t*} indicate the corresponding true unknown longitudinal profile up to time t. Therefore, the PHs time-to-event sub-model is given by [26, 27]:

$$h(t|M_{i}(t),u_{i})=h_{0}(t)exp[\psi^{\prime }u_{i}+\rho m_{i}(t)]$$

where ***h***_**0**_(***t***) is the baseline hazard function, and ***u***_***i***_∈***U***_***i***_ is the set of time-invariant variables, *ψ* indicates the associated vector of log hazard ratios, ***ρ*** indicates the association parameter. The exp (*ρ*) quantifies the hazard ratio for a one-unit increase in ***m***_***i***_(***t***), at time ***t***. Including the true unobserved trajectory function, ***m***_***i***_(***t***), into the linear predictor of the PHs model provides a way to link the component sub-models to form the joint modeling framework. It is used to combine the longitudinal with the survival sub-model.

There are three common association structures; “current value”, “current value and slope”, and “shared random effects” parameterization [13, 28–30]. Among those association parameterizations the “current value and slope” was used, hence our interest is to see the effects of the current true values and slopes of BPs on first remission time of hypertensive patients.

## Results

Data exploration was done using tabular and graphical approaches. Among 301 hypertensive patients involved this study, 153 (50.8%) were male, and 124 (49.2%) were residents from rural areas (see Table 2). About 83(27.6%), 58 (19.3%), 82 (27.2%), and 25 (8.3%) had a history of diabetes mellitus, CKD, stroke, and HIV respectively. The median time to first remission was 11 months. About 123 (50.6%) of the 243 hypertensive patients having the first remission were male and 99 (40.7%) resided in the rural area. The mean SBP and DBP of hypertensive patients with their corresponding standards were 152.18 (18.68) mmHg and 92.79 (11.92) mmHg respectively. History of DM and stroke were significantly associated with time to first remission.

**Table 2:** Frequency distribution for baseline independent variables together with the censored observations of time-to-first remission and their association.

Variables	Categories	Censored (%)	Event (%)	Total (%)	*X*^2^ (*p*−*value*)
Sex	Male	30 (9.9)	123 (40.9)	153 (50.8)	2.85e^-05^ (0.996)
	Female (Ref)	28 (9.3)	120 (39.9)	148 (49.2)	
Residence	Urban	33 (11.0)	144 (47.8)	177 (58.8)	0.0324 (0.857)
	Rural (Ref)	25 (8.3)	99 (32.9)	124 (41.2)	
History of DM	Yes	30 (10.0)	53 (17.6)	83 (27.6)	19.509 **(<0.001)**
	No (Ref)	28 (9.3)	190 (63.12)	218 (72.4)	
History of CKD	Yes	16 (5.3)	42 (14.0)	58 (19.3)	2.5668 (0.109)
	No (Ref)	42 (14.0)	201 (66.8)	243 (80.8)	
History of stroke	Yes	25 (8.3)	57 (18.9)	82 (27.2)	8.1542 **(0.004)**
	No (Ref)	33 (11.0)	186 (61.8)	219 (72.8)	
History of HIV	Yes	9 (3.0)	16 (5.3)	25 (8.3)	3.8032 (0.051)
	No (Ref)	49 (16.3)	227 (75.4)	276 (91.7)	
Regimen	Monotherapy (Ref)	6 (2.0)	32 (10.6)	38 (12.6)	0.3439 (0.842)
	Two drug therapy	20 (6.6)	80 (26.6)	100 (33.2)	
	Three or more drug therapy	32 (10.6)	131 (43.5)	163 (54.1)	
Drug type	Enalapril (Ref.)	20 (6.6)	83 (27.6)	103 (34.2)	2.4682 (0.481)
	Nefidipine	13 (4.3)	72 (23.9)	85 (28.2)	
	Enalapril + Nefidipine	19 (6.3)	58(19.3)	77 (25.6)	
	Others	6 (2.0)	30 (10.0)	36 (12.0)	
Total (%)		58 (19.3)	243 (80.7)	301 (100)	

Ref. indicates the reference category

At the baseline, the average age of hypertensive patients with its corresponding standard deviation was 51.77 (Sd. = 13.88) years, and the mean SBP and DBP of hypertensive patients with their corresponding standard were 152.18 (Sd. = 18.68) mmHg and 92.79 (Sd. = 11.92) mmHg respectively.

**Fig 1:**
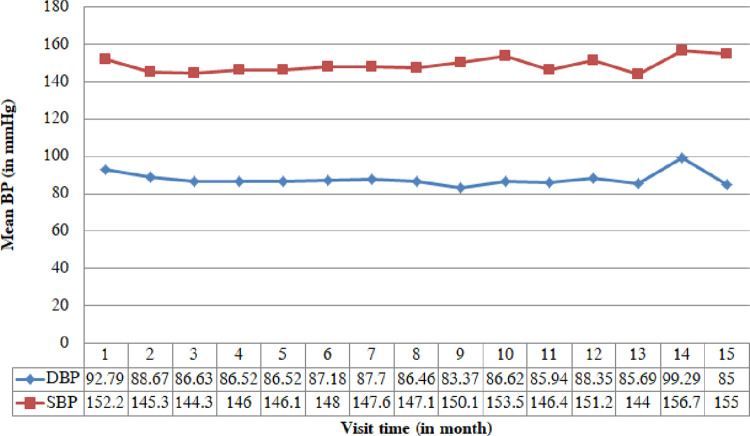
The average BPs plot over time.

The average progress of both SBP and DBP was revealed in Fig 1. Both BPs look in a constant pattern throughout entire visit times. The mean SBP varies between 144 and 156.71 mmHg while the mean DBP varies between 83.37 and 99.29 mmHg.

**Fig 2:**
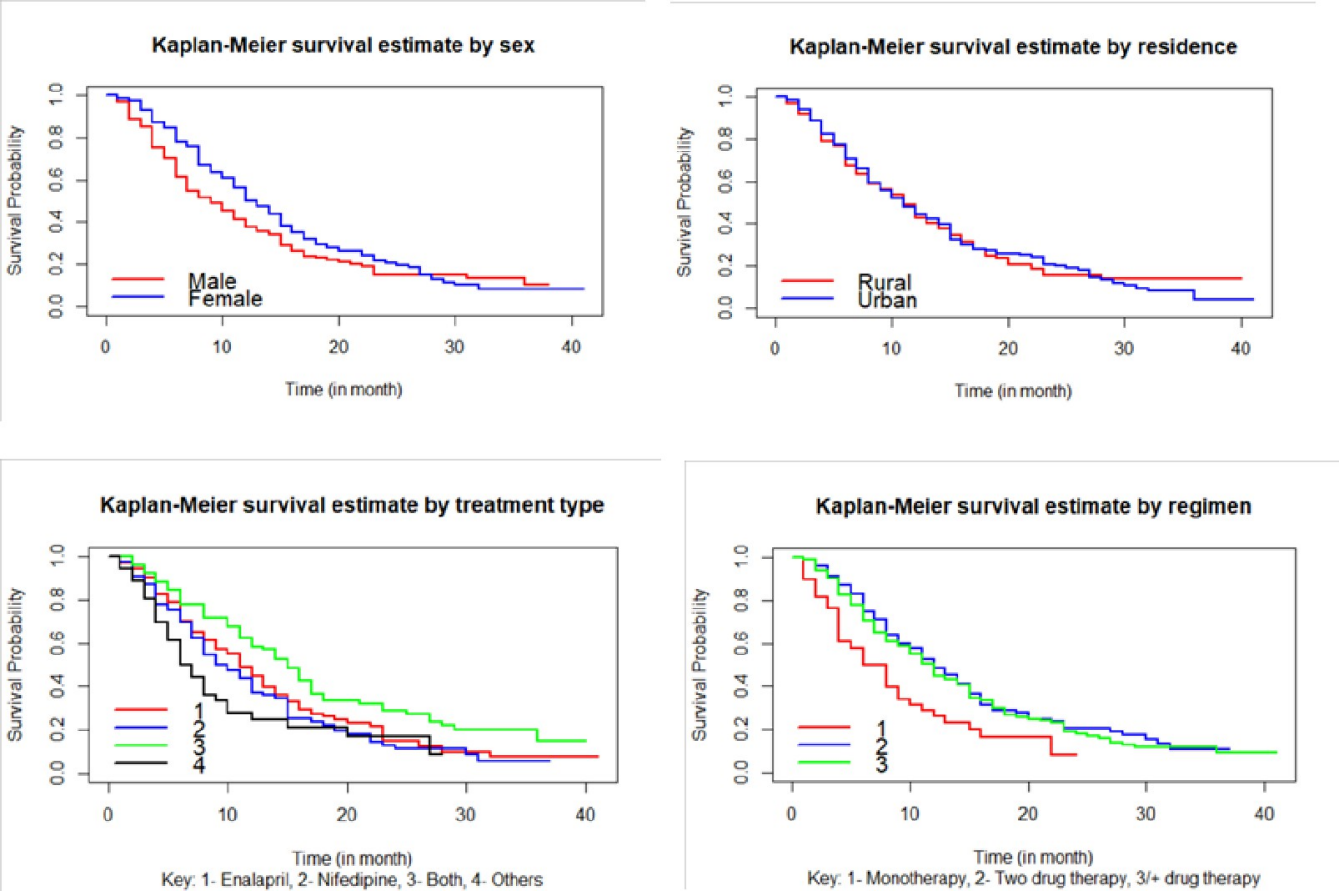
Kaplan-Meier plot for the survival function sex, residence, treatment type, and regimen.

The Kaplan–Meier survival curves in Fig 2 revealed that the time to the first remission of male subjects was faster as compared to females.

**Fig 3:**
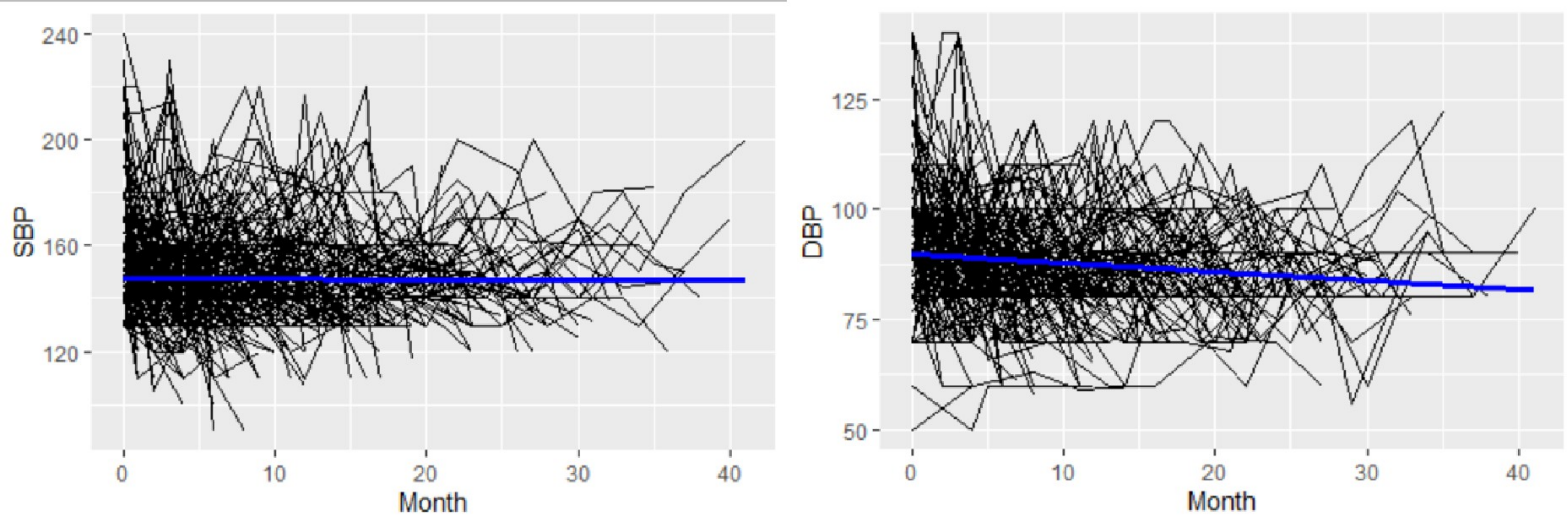
Individual profiles with average trend line for SBP and DBP.

The profile plot in Fig 3 depicts that the patients began with a varying baseline of SBP and DBP. It also shows the progress of SBP and DBP are different over time. The mean profile shows patients’ SBP stays relatively constant during the research period, while their DBP declines with time.

The estimated standard deviation of random intercepts of SBP and DBP was 10.8131 and 6.7399 respectively, which is larger than the estimated standard deviation of the random slope of SBP 0.8605 and DBP 0.5223. This indicates that between-subjects variability at baseline is larger than longitudinal trajectories.

The evolution of association was used to find the marginal correlation between the two responses SBP and DBP at different visit times. i.e., for the first three visit times, the marginal correlation between the two BPs was 0.260 (at the first visit), 0.246 (at the second visit), and 0.244 (at the third visit, which shows a little decrement.

As the patient’s BUN level increases by a unit mg/dl the average SPB and DBP of the patient will be increased by 0.06 mmHg (se = 0.0007) and 0.03 mmHg (0.0004) respectively (see Table 3). The average SBP and DBP of patients had a 4.97 mmHg and 2.39 mmHg increment for patients who had a history of CKD than those patients who had no history of CKD. The time to attain the first remission for patients who had a history of diabetes mellitus was 46% lower than those who had no history of diabetes mellitus.

**Table 3:** Bayesian joint model parameter estimates for longitudinal and survival processes.

**Multivariate linear mixed-effects sub-model**					
S/No.	Variables	Post Mean	SE	95% CI	
				Lower	Upper
Fixed effect: SBP					
1.	Intercept	96.9114	0.1659	85.3081	107.9613
2.	Obstime	-0.8399	0.0056	-1.0784	-0.6177
3.	Age	0.3791	0.0018	0.2804	0.4797
4.	Residence (Urban)	4.2451	0.0544	1.0868	7.2590
5.	BCL	0.0139	0.0005	-0.0128	0.0404
6.	BUN	0.0551	0.0007	0.0139	0.0947
7.	Creatinine	-0.6129	0.0191	-1.7826	0.5991
8.	Calcium	1.9366	0.0243	0.4064	3.4828
9.	Hemoglobin	1.2274	0.0093	0.6672	1.8179
10.	DM (Yes)	4.3695	0.0442	1.7936	6.9780
11.	CKD (Yes)	4.9710	0.0566	1.1341	9.0353
12.	Stroke (Yes)	2.4524	0.0432	-0.1896	5.1582
13.	HIV (Yes)	2.0087	0.0805	-2.8333	6.6961
14.	Nifedipine	1.8783	0.0611	-1.9193	5.6376
	Both	6.9160	0.0621	3.0214	10.6390
	Others	0.6605	0.0820	-3.9206	5.2810
DBP					
15.	Intercept	36.7942	0.2725	21.3154	52.5219
16.	Obstime	-0.6199	0.0033	-0.7695	-0.4853
17.	Age	0.0453	0.0009	-0.0182	0.1047
18.	Residence (Urban)	1.2731	0.0299	-0.6304	3.0068
19.	BCL	0.0090	0.0003	-0.0078	0.0259
20.	BUN	0.0319	0.0004	0.0071	0.0560
21.	Calcium	0.5437	0.0141	-0.3214	1.4333
22.	Sodium	0.1856	0.0018	0.0809	0.2999
23.	Chlorine	0.0932	0.0019	-0.0178	0.2148
24.	Hemoglobin	0.6380	0.0058	0.2874	0.9996
25.	CKD (Yes)	2.3889	0.0345	0.2789	4.4804
26.	HIV (Yes)	1.6150	0.0474	-1.2648	4.4665
27.	Nifedipine	1.8687	0.0356	-0.3455	3.9687
	Both	3.6535	0.0362	1.5597	5.7307
	Others	0.6838	0.0495	-2.2906	3.7647
Random effect					
S/No.	Coefficient	Std. dev (95% CI).	Corr.		
28.	*α*_*i*1_	10.8131 (6.1020, 14.0850)	*α*_*i*1_		
29.	*b*_*i*1_	0.8605 (0.2456, 1.8974)	-0.3422	*b*_*i*1_	
30.	*α*_*i*3_	6.7399 (2.6981, 9.6325)	0.7720	-0.3151	*α*_*i*2_
31.	*b*_*i*2_	0.5223 (0.1879, 0.9354)	-0.4394	0.9398	-0.4257
32.	*ε*_*i*1_	15.0348	0.0094	14.4514	15.6740
33.	*ε*_*i*2_	9.5268	0.0060	9.1777	9.9037

S/No.	Variables	Categories	Estimate	SE	HR	95% CI	
						Lower	Upper
34.	Age	-	-0.0013	0.0001	0.9987	0.9893	0.9992
35	Strata (Sex)	Male	-0.4635	0.0063	0.6291	0.4611	0.8533
36.	BUN	-	-0.0005	0.0000	0.9995	0.9981	1.0031
37.	Regimen	Two drug therapy	-0.3714	0.0071	0.6898	0.4205	1.1146
		3 or more therapy	-0.1016	0.0071	0.9034	0.5455	1.4715
38.	Comorbidity	-	-0.1984	0.0035	0.8200	0.9740	1.5050
39.	DM	Yes	-0.6197	0.0066	0.5381	0.3558	0.8165
40.	CKD	Yes	-1.0951	0.0075	0.3345	0.2158	0.5203
41.	Strata (Stroke)	Yes	-0.6819	0.0058	0.5057	0.3520	0.7255
42.	Strata(HIV)	Yes	-0.2793	0.0090	0.7563	0.4284	1.2911
43.	Treatment type	Nifedipine	-0.0824	0.0057	0.9209	0.6484	1.3111
		Both	-0.0718	0.0065	0.9307	0.6229	1.3723
		Others	0.5650	0.0077	1.7594	1.1009	2.7991
Association parameter							
44.	*ρα*_*SBP*_	-	-0.1128	0.0007	0.8933	0.8616	0.9241
45.	*ρb*_*SBP*_	-	-1.3218	0.0082	0.2667	0.1579	0.4247
46.	*ρα*_*DBP*_	-	-0.0133	0.0011	0.9868	0.9344	1.0396
47.	*ρb*_*DBP*_	-	-0.0024	0.0000	0.9976	0.9951	1.0002

Key: **ρ**^α^SBP and ρ^b^SBP the association parameter for the current true value and slope of SBP respectively; ρ^α^DBP and ρ^b^DBP the association parameter for the current true value and slope of DBP respectively. αi and bi corresponds with random intercept and random slope.

The hazard of the patient’s first remission time for males was 0.63 times less likely than the hazard for females (see Table 3). The time to attain the first remission for patients who had taken other types of treatment was 76% higher than for those who had taken enalapril. The estimate of the association parameter for the current true value of SBP (*ρα*_*SBP*_) was −0.1128 (*HR* = 0.89 (0.86 to 0.92)); there is a 0.89-fold decrease in risk of the first remission, per doubling of SBP. i.e., for a unit mmHg increase on the SBP the rate of first remission time of patients will be decreased by 10.67%.

## Discussion

This study attempted to jointly model the longitudinal change of blood pressures (SBP and DBP) and time to the first remission of hypertensive outpatients receiving treatment. The data were explored using several approaches such as mean plot, profile plot, and Kaplan-mier estimates. To estimate the effects of the socioeconomic, demographic and biological characteristics joint longitudinal and survival models using the Bayesian approach were employed.

According to the results of the study conducted at Jimma University Specialized Hospital, Ethiopia, there was a high correlation between BPs evolution and the association of evolution approximates one [2, 31]. The evolution of association between the BPs at the baseline was 0.260 and has a slight increment as time changes, this is also supported by a study FHRH, Bahir Dar, Ethiopia [10]. In the multivariate longitudinal sub-model; the observation time was negatively associated with the average SBP and DBP, that is, as the observation time increases patient’s average SBP and DBP had increased proportionately. Age also had a statistically significant association with the average SBP. This report was consistent with reports done at Jimma University specialized hospital, Ethiopia [31], and India [32].

The majority of hypertension patients (80.7%) experienced a brief period of disease remission, but later, their blood pressure may rise again. It was greater than other studies conducted at Adama Hospital Medical College Ethiopia (43.6%) [33], northwest Ethiopia (42.9%) [34], urban-rural China (45.9%) [35], Asian-Indians (48.7%) [36], Macau-China (49%) [37], Sudanese adults (64%) [38], and many other studies done in the world. This discrepancy may be due to different lifestyles and eating habits, awareness of the need for blood pressure control, education and communication strategies, as well as degrees of clinical and pharmaceutical adherence. Age was the significant factor for the first remission time of hypertensive patients that for a year increase on their age, the hazard of achieving the first remission for patients was decreased by 0.13% (*HR* = 0.9987).

The patient’s residence area was significantly associated with only the average SBP, not with the average DBP. This finding was in line with a study in [31]. Patients who had a history of DM had a 4.37 mmHg increment on their average SBP than those patients who had no history of DM. The previous community-based cross-sectional study conducted among adults in Ethiopia corroborates this finding [39], which was supported by a study done in northwest Ethiopia [6]. Compared to individuals who were in a smaller age range, elderly patients had a lower chance of experiencing their initial remission. This conclusion is supported by research done at Bahir-Dar Felege Hiwot Referral Hospital. [11]. However, it was not supported by the study conducted in northwest Ethiopia [34], this may be due to environmental variation and the physical activity trend of patients.

According to research done in northwest Ethiopia [34], female patients’ first remission times were longer than male patients’ in line with this study. Females have had the first remission more frequently than males in middle- and low-income nations [36], Bangladesh [40], self-selected sub-Saharan African urban population [41], Nsukka, Nigeria [42], and Sudanese adults [38]. Patients who had a history of diabetes mellitus and CKD had poor controlling BP than patients who had no history of diabetes mellitus and CKD. This is following the research conducted in South Asia and China [43, 44], northwest Ethiopia [34], and Ayder comprehensive specialized hospital, Tigray, Ethiopia [45].

The posterior estimates of the association parameters in the joint analysis are significantly different from zero, confirming that the two sub-models are associated and in line with research using the Bayesian joint model, even though the estimated parameters of the separate longitudinal model and the longitudinal sub-model under the joint model are exactly equal [46]. In the end, the Bayesian joint longitudinal-survival model; that the longitudinal outcomes are associated with the survival one, minimizes the computing constraints and brings more consistent parameter estimates. Thus, health professionals and policymakers need to incorporate the findings of this study for their short and long-run strategies. The authors also would like future researchers to refer to these results on related issues.

## Conclusion

Patients receiving both nifedipine and enalapril treatments have significantly higher SBP and DBP compared to patients receiving only enalapril, which may translate into a lower risk of first remission through the association. On the other hand, there is no evidence of a significant difference in SBP and DBP levels for patients receiving "other" treatments compared to patients receiving only enalapril in the longitudinal models but their risk of first remission is significantly increased. Therefore both the longitudinal and survival sub-models bring some piece of information about the treatments effect, which justifies the use of a joint model. The patients who had a good follow-up, lower BUN, lower serum calcium, lower serum sodium, lower hemoglobin, and take the treatment enalapril showed an opportunity in decreasing their blood pressure. This compels patients to experience the first remission early. Besides, age, patient’s history of DM, patient’s history of CKD, and treatment type were the joint determinants of longitudinal change of blood pressure and the first remission time. The findings of this study are supportive for the health authorities and legislators, and it will help as a reference for other researchers.

## Abbreviations

AFT: Accelerated Failure Time
AIC: Akaike Information Criterion
BP: Blood Pressure
CKD: Chronic Kidney Disease
CVD: Coronary Vascular Disease
DBP: Diastolic Blood Pressure
SBP: Systolic Blood Pressure

## References

[1] Organization, W.H., Global health risks: mortality and burden of disease attributable to selected major risks. 2009: World Health Organization.

[2] Mogi M., et al., Annual reports on hypertension research 2020. Hypertension Research, 2022. 45(1): p. 15–31. 10.1038/s41440-021-00766-3 34650193

[3] Organization, W.H., P.H.A.o. Canada, and C.P.H.A.o. Canada, Preventing chronic diseases: a vital investment. 2005: World Health Organization.

[4] Petrakis V., et al., Diabetes mellitus and hypertension as major risk factors of mortality from Covid-19 pneumonia. Experimental and Clinical Endocrinology & Diabetes, 2022. 130(03): p. 205–206. doi: 10.1055/a-1325-0381 33296923

[5] Kearney P.M., et al., Global burden of hypertension: analysis of worldwide data. The lancet, 2005. 365(9455): p. 217–223. doi: 10.1016/S0140-6736(05)17741-1 15652604

[6] Awoke A., et al., Prevalence and associated factors of hypertension among adults in Gondar, Northwest Ethiopia: a community based cross-sectional study. BMC Cardiovascular Disorders, 2012. 12(1): p. 113. doi: 10.1186/1471-2261-12-113 23186560 PMC3519757

[7] Tesfaye F., Byass P., and Wall S., Population based prevalence of high blood pressure among adults in Addis Ababa: uncovering a silent epidemic. BMC Cardiovascular Disorders, 2009. 9(1): p. 39. 19698178 10.1186/1471-2261-9-39 PMC2736927

[8] Fuchs F.D. and Whelton P.K., High blood pressure and cardiovascular disease. Hypertension, 2020. 75(2): p. 285–292. 31865786 10.1161/HYPERTENSIONAHA.119.14240 PMC10243231

[9] Choi Y.-H., Chowdhury R., and Swaminathan B. Prediction of hypertension based on the genetic analysis of longitudinal phenotypes: a comparison of different modeling approaches for the binary trait of hypertension. in BMC proceedings. 2014. BioMed Central. 10.1186/1753-6561-8-S1-S78 PMC4143688 25519406

[10] Workie D.L., Zike D.T., and Fenta H.M., Bivariate longitudinal data analysis: a case of hypertensive patients at Felege Hiwot Referral Hospital, Bahir Dar, Ethiopia. BMC Research Notes, 2017. 10(1): p. 722. 29221495 10.1186/s13104-017-3044-4 PMC5721485

[11] Sendek E.M. and Hebo S.H., Modeling time-to-good control of hypertension using Cox proportional hazard and frailty models at Bahir-Dar Felege Hiwot Referral Hospital. Open Access Medical Statistics, 2017. 7: p. 27–36.

[12] Webb A.J. and Werring D.J., New insights into cerebrovascular pathophysiology and hypertension. Stroke, 2022. 53(4): p. 1054–1064. 35255709 10.1161/STROKEAHA.121.035850 PMC7615037

[13] Lawrence Gould A., et al., Joint modeling of survival and longitudinal non‐survival data: current methods and issues. Report of the DIA Bayesian joint modeling working group. Statistics in medicine, 2015. 34(14): p. 2181–2195. 24634327 10.1002/sim.6141 PMC4677775

[14] Papageorgiou G., et al., An overview of joint modeling of time-to-event and longitudinal outcomes. Annual review of statistics and its application, 2019.

[15] Khoundabi B., et al., Acute kidney injury risk factors for icu patients following cardiac surgery: The application of joint modeling. Trauma Monthly, 2016. 21(4). 10.5812/traumamon.23749 PMC5282936 28180122

[16] Bitew M., Tafere A., and Tolosa T., Study on bovine mastitis in dairy farms of Bahir Dar and its environs. Journal of Animal and Veterinary Advances, 2010. 9(23): p. 2912–2917.

[17] Cox D.R. and Snell E.J., A general definition of residuals. Journal of the Royal Statistical Society: Series B (Methodological), 1968. 30(2): p. 248–265.

[18] Animut Y., Assefa A.T., and Lemma D.G., Blood pressure control status and associated factors among adult hypertensive patients on outpatient follow-up at University of Gondar Referral Hospital, northwest Ethiopia: a retrospective follow-up study. Integrated blood pressure control, 2018. 11: p. 37. 29720880 10.2147/IBPC.S150628 PMC5918628

[19] Schiavon C.A., et al., Effects of Bariatric Surgery in Obese Patients With Hypertension: The GATEWAY Randomized Trial (Gastric Bypass to Treat Obese Patients With Steady Hypertension). Circulation, 2018. 137(11): p. 1132–1142. 29133606 10.1161/CIRCULATIONAHA.117.032130 PMC5865494

[20] Ibrahim J.G., Chen M.H., and Sinha D., B ayesian Survival Analysis. Wiley StatsRef: Statistics Reference Online, 2014.

[21] Zhang D., et al., Assessing model fit in joint models of longitudinal and survival data with applications to cancer clinical trials. Statistics in Medicine, 2014. 33(27): p. 4715–4733. 25044061 10.1002/sim.6269 PMC4221436

[22] Brookhart M.A., et al., Variable selection for propensity score models. American journal of epidemiology, 2006. 163(12): p. 1149–1156. 16624967 10.1093/aje/kwj149 PMC1513192

[23] Buta G.B., Goshu A.T., and Worku H.M., Bayesian joint modelling of disease progression marker and time to death event of HIV/AIDS patients under ART follow-up. Journal of Advances in Medicine and Medical Research, 2015: p. 1034–1043.

[24] Erango M.A., et al., Bayesian joint modelling of survival of HIV/AIDS patients using accelerated failure time data and longitudinal CD4 cell counts. Br J Med Med Res, 2017. 20(6): p. 1–12.

[25] Diggle P., et al., Analysis of longitudinal data. 2002: Oxford University Press.

[26] Hsieh F., Tseng Y.K., and Wang J.L., Joint modeling of survival and longitudinal data: likelihood approach revisited. Biometrics, 2006. 62(4): p. 1037–1043. 17156277 10.1111/j.1541-0420.2006.00570.x

[27] Cox D.R. and Oakes D., Analysis of survival data. Vol. 21. 1984: CRC Press.

[28] Cekic S., et al., A tutorial for joint modeling of longitudinal and time-to-event data in R. arXiv preprint arXiv:1909.05661, 2019.

[29] Rizopoulos D., Joint models for longitudinal and time-to-event data: With applications in R. 2012: CRC press.

[30] Rizopoulos D., et al., Combining dynamic predictions from joint models for longitudinal and time-to-event data using Bayesian model averaging. Journal of the American Statistical Association, 2014. 109(508): p. 1385–1397.

[31] Negash Y., Kassahun W., and Gurmassa A., Joint Modeling of Longitudinal Systolic and Diastolic Blood Pressure Measurements of Hypertensive Patients Receiving Treatment in Jimma University Specialized Hospital. 2014.

[32] Wang W., et al., A longitudinal study of hypertension risk factors and their relation to cardiovascular disease: the Strong Heart Study. Hypertension, 2006. 47(3): p. 403–409. 16432042 10.1161/01.HYP.0000200710.29498.80

[33] Lichisa G., et al., Blood pressure control and its contributing factor among ambulatory hypertensive patients in Adama Hospital medical college, East Shoa, Adama, Ethiopia. Int J Pharm Biol Sci Res Dev, 2014. 2(7): p. 1–15.

[34] Teshome D.F., Demssie A.F., and Zeleke B.M., Determinants of blood pressure control amongst hypertensive patients in Northwest Ethiopia. PloS one, 2018. 13(5): p. e0196535. 29718964 10.1371/journal.pone.0196535 PMC5931630

[35] Mohan V., et al., Prevalence, awareness and control of hypertension in Chennai-the Chennai Urban Rural Epidemiology Study (CURES-52). Journal of the Association of Physicians of India, 2007. 55: p. 326–332. 17844691

[36] Chow C.K., et al., Prevalence, awareness, treatment, and control of hypertension in rural and urban communities in high-, middle-, and low-income countries. Jama, 2013. 310(9): p. 959–968. 24002282 10.1001/jama.2013.184182

[37] Ke L., et al., Prevalence, awareness, treatment and control of hypertension in Macau: results from a cross-sectional epidemiological study in Macau, China. American journal of hypertension, 2015. 28(2): p. 159–165. 25063734 10.1093/ajh/hpu121

[38] Babiker F.A., Elkhalifa L.A., and Moukhyer M.E., Awareness of hypertension and factors associated with uncontrolled hypertension in Sudanese adults: cardiovascular topic. Cardiovascular journal of Africa, 2013. 24(6): p. 208–212. 24217260 10.5830/CVJA-2013-035 PMC3767941

[39] Asresahegn H., Tadesse F., and Beyene E., Prevalence and associated factors of hypertension among adults in Ethiopia: a community based cross-sectional study. BMC Research Notes, 2017. 10(1): p. 629. 29183367 10.1186/s13104-017-2966-1 PMC5704552

[40] Rahman M.M., et al., Prevalence and control of hypertension in Bangladesh: a multilevel analysis of a nationwide population-based survey. Journal of hypertension, 2015. 33(3): p. 465–472. 25380166 10.1097/HJH.0000000000000421

[41] Dzudie A., et al., Prevalence, awareness, treatment and control of hypertension in a self-selected sub-Saharan African urban population: a cross-sectional study. BMJ open, 2012. 2(4). 10.1136/bmjopen-2012-001217 PMC3433777 22923629

[42] Ekwunife O.I., Udeogaranya P.O., and Nwatu I.L., Prevalence, awareness, treatment and control of hypertension in a Nigerian population. 2010.

[43] Jafar T.H., et al., Determinants of uncontrolled hypertension in rural communities in South Asia—Bangladesh, Pakistan, and Sri Lanka. American journal of hypertension, 2018. 31(11): p. 1205–1214. 29701801 10.1093/ajh/hpy071 PMC6188532

[44] Choudhary R., et al., Awareness, treatment adherence and risk predictors of uncontrolled hypertension at a tertiary care teaching hospital in Western India. Indian Heart J, 2016. 68(Suppl 2): p. S251. 27751307 10.1016/j.ihj.2016.08.003 PMC5067811

[45] Gebremichael G.B., Berhe K.K., and Zemichael T.M., Uncontrolled hypertension and associated factors among adult hypertensive patients in Ayder comprehensive specialized hospital, Tigray, Ethiopia, 2018. BMC cardiovascular disorders, 2019. 19(1): p. 1–10. 31117945 10.1186/s12872-019-1091-6 PMC6532230

[46] Seid A., et al., Joint modeling of longitudinal CD4 cell counts and time-to-default from HAART treatment: a comparison of separate and joint models. Electronic Journal of Applied Statistical Analysis, 2014. 7(2): p. 292–314.

